# Constitutive activation of the ETS-1-miR-222 circuitry in metastatic melanoma

**DOI:** 10.1111/j.1755-148X.2011.00881.x

**Published:** 2011-06-28

**Authors:** Gianfranco Mattia, M Cristina Errico, Federica Felicetti, Marina Petrini, Lisabianca Bottero, Luisa Tomasello, Paolo Romania, Alessandra Boe, Patrizia Segnalini, Antonio Di Virgilio, Mario P Colombo, Alessandra Carè

**Affiliations:** 1 Department of Hematology, Oncology and Molecular Medicine, Istituto Superiore SanitàRome, Italy; 2 Service for Quality and Safety of Animal Experimentation, Istituto Superiore di SanitàRome, Italy; 3 Immunotherapy and Gene Therapy Unit, Department of Experimental Oncology, Fondazione IRCCS Istituto Nazionale TumoriMilan, Italy

**Keywords:** ETS-1, melanoma, microRNA-222, tumor progression

## Abstract

MicroRNAs-221 and -222 are highly upregulated in several solid tumors, including melanomas. We demonstrate that the proto-oncogene ETS-1, involved in the pathogenesis of cancers of different origin, is a transcriptional regulator of miR-222 by direct binding to its promoter region. Differently from 293FT cells or early stage melanomas, where unphosphorylated ETS-1 represses miR-222 transcription, in metastatic melanoma the constitutively Thr-38 phosphorylated fraction of ETS-1 induces miR-222. Despite its stepwise decreased expression along with melanoma progression, the oncogenic activity of ETS-1 relies on its RAS/RAF/ERK-dependent phosphorylation status more than on its total amount. To close the loop, we demonstrate ETS-1 as a direct target of miR-222, but not miR-221, showing the novel option of their uncoupled functions. In addition, a spatial redistribution of ETS-1 protein from the nucleus to the cytoplasm is also evidenced in advanced melanoma cells. Finally, in vivo studies confirmed the contribution of miR-222 to the increased invasive potential obtained by ETS- silencing.

## Introduction

Cutaneous melanoma is one of the most aggressive neoplasms. Although surgical excision is mostly a definitive treatment at the early stages of the disease, at present, standard treatments are ineffective after metastatic dissemination (Chudnovsky et al., 2005; Gray-Schopfer et al., 2007). The identification of new suitable prognostic and diagnostic markers and possibly therapeutic targets has been thoroughly researched (Gremel et al., 2009).

MicroRNAs (miRNAs) are a family of small, non-coding RNAs able to post-transcriptionally repress gene expression by pairing to the 3′UTR of target mRNAs (Calin and Croce, 2006). MiRNAs are involved in all the main biological processes and their aberrant deregulated expression has functional implications in tumor development (Inui et al., 2010). The miR-221 and -222 have been described in several types of cancer and consistently in melanoma (Felicetti et al., 2008; le Sage et al., 2007; Medina et al., 2008). Indeed, they enhance tumorigenicity in non-small cell lung cancer (NSCLC) and hepatocarcinoma cells (HCC) by targeting PTEN and TIMP3 tumor suppressors; in turn, they are activated by c-MET through the AP-1 transcription complex (Garofalo et al., 2009). We have reported that miR-221 and -222 act on melanoma progression through multiple oncogenic pathways downregulating p27Kip1 and c-KIT receptors, leading to enhanced proliferation and blocking the differentiation of melanoma cells (Felicetti et al., 2008).

**Significance**

MiR-221 and -222 have been reported as important regulators of tumor cell proliferation, migration and invasion in cancers of different origins, including melanoma. Growing evidence suggests their inhibition as a promising option for a novel therapeutic approach, particularly relevant in melanoma still lacking successful treatments. However, a complete knowledge of miR-221/-222 actual targets and downstream pathways is obligatory before opening this door. Here, we reveal the existence of a novel ETS-1↔miR-222 circuitry in which both miR-222 and the phosphorylated activating portion of ETS-1 are relevant to melanoma progression.

Looking for new miR-221/-222-dependent target genes, we focused on the proto-oncogene ETS-1, the founding member of the family of ETS transcriptional factors known to be involved in the pathogenesis of cancers of different origin. However, the role of ETS-1 in melanoma is far from being clearly demonstrated.

ETS-1 protein regulates many target genes by functionally or physically interacting with several transcription factors whose combinations lead to either gene activation or repression (Dittmer, 2003). ETS-1 p51 protein binds to purine-rich DNA sequence, containing a conserved GGAA/T core sequence, through its DNA binding domain. ETS-1 is also post-translationally regulated in that RAS increases its transcriptional activity through ERK1/2 (Yang et al., 1996), which phosphorylates ETS-1 at a single threonine (T38) within the N-terminal domain (Seidel and Graves, 2002). Conversely, CaMKII is able to mediate calcium-dependent inactivation of ETS-1 DNA binding by phosphorylating the regulatory exon VII domain of ETS-1 (Cowley and Graves, 2000; Pognonec et al., 1988).

We selected ETS-1 among many putative miR-221/-222-regulated target genes based on the good score reported for the main specific sites using TargetScan, microRNA and PicTar, which have been specifically developed for the identification of the actual targets of microRNA. Besides that, and possibly more importantly, we focused on the functional role of ETS1, bearing in mind that in melanoma, basic fibroblast growth factor (bFGF) constitutive activation is the main initiating factor of the MAPK-ERK-Ets1/2 signaling cascade (Carè et al., 1996; Squarzoni et al., 2011). ETS-1 is expressed in melanocytes migrating from the neural crest during embryogenesis and in normal adult melanocytes, but its role in malignant melanomas appears controversial. ETS-1 has been reported either as a valuable diagnostic/prognostic marker (Rothhammer et al., 2004) or as molecule with no clear association with clinical outcome (Torlakovic et al., 2004).

Here we demonstrate the existence of an ETS-1↔miR-222 circuitry relevant to melanoma progression. We identify ETS-1 as a transcriptional regulator of miR-222, by direct binding to its promoter region, showing its functional shift, from negative to positive regulator, in early and advanced melanoma cells, respectively. Unlike low grade malignant cells, where ETS-1 represses miR-222 transcription, in metastatic melanomas ETS-1 and miR-222 appear directly interconnected by a deregulated feedback loop whereby the persistent activation of the MAPK-ERK1/2 cascade leads to Thr-38 phosphorylation of ETS-1 and miR-222 induction, in turn increasing proliferation and tumor malignancy (Felicetti et al., 2008). In addition, we show that ETS-1 is directly targeted by miR-222, but not by miR-221, indicating the option of miR-221 and -222 uncoupled functionalities. Considering the role of c-JUN in miR-221/-222 activation (Garofalo et al., 2009), we suggest an ETS-1/c-JUN collaboration where ETS-1 seems to fine tune miR-222 as well as c-JUN itself (Goldberg et al., 1994). Finally, based on our previous results showing the tumor-suppressor role of the promyelocytic leukemia zinc finger (PLZF) product in melanoma, we examined the possibility of an ETS-1/PLZF coordinated function.

## Results

### ETS-1 expression in melanoma

**Figure 1:**
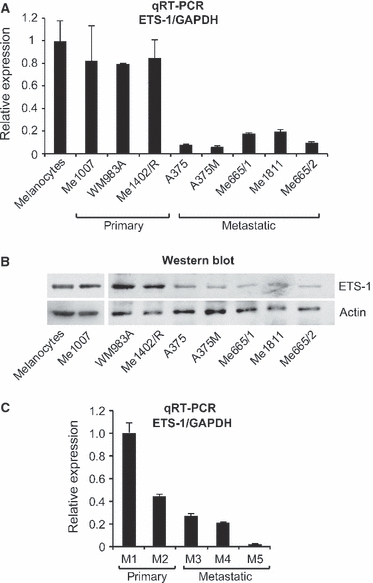
ETS-1 expression in normal human melanocytes and melanomas. (A) qRT-PCR and (B) Western blot analyses of stabilized melanoma cell lines at different stage of progression. A representative WB is shown. Normal human melanocytes are included as a normal reference and actin as a housekeeping loading control. (C) qRTPCR analysis of melanomas at early in vitro passages. qRT-PCR data represent the average ± SD of at least three separate experiments.

We evaluated ETS-1 expression in a panel of melanoma cell lines at different stages of progression, including primary vertical growth phase (VGP) melanomas, as well as subcutaneous and lymph-node metastases (Felicetti et al., 2004). Traditional and qRT-PCR as well as Western blot analyses on nuclear extracts showed that all these melanoma cell lines expressed ETS-1 at levels inversely related to melanoma malignancy. In particular, primary and metastatic melanomas expressed high and low amounts of ETS-1 at mRNA (Figure 1A) and protein levels (Figure 1B), respectively. To rule out any possible artifactual result due to in vitro cell culture, this expression pattern was confirmed in early passage cells obtained from melanoma bioptic samples (Figure 1C). Note that a high level of ETS-1 mRNA and protein was detected in normal human melanocytes (Figure 1A).

### ETS-1 phosphorylation

**Figure 2:**
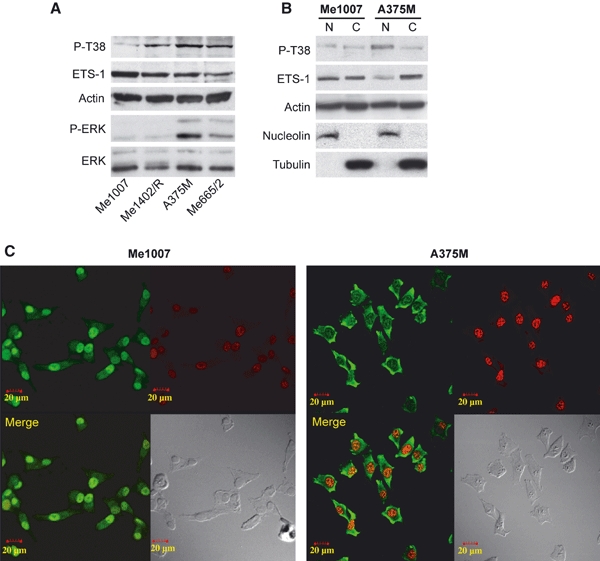
Analysis of ETS-1 phosphorylation at Thr-38. (A) Representative Western blot of ETS-1 and its P-T38-fraction, ERK1/2 and P-ERK1/2 in four differently staged melanoma cell lines. (B) Nuclear and cytoplasmic extracts of Me1007 and A375M, shown as primary and metastatic representative melanomas. Actin, nucleolin and tubulin have been included as loading controls. (C) Confocal immunofluorescence analysis with anti-ETS-1 (green staining) and anti-P-T38-ETS-1 (red staining) antibodies assessing the different levels and distribution of total ETS-1 and T-38 phosphorylated fraction in the primary Me1007 and metastatic A375M melanoma cell lines.

Considering that the transcription factor ETS-1 regulates many tumor-promoting factors, we reasoned that its downregulation along with melanoma progression cannot explain its presumed oncogenic role, which might rather correlate with an increased activity due to anomalous post-translational modifications. ETS-1 molecules harbor two phosphorylation sites, threonine-38 (T38) and an array of serines within the exon VII domain (4S: S251, S257, S282, S285). Phosphorylation at T38 by ERK1/2 activates ETS-1, whereas phosphorylation of the exon VII 4S domain by CaMKII or MLCK reduces ETS-1 DNA binding and transcriptional activity (Dittmer, 2003). To test our hypothesis, we analyzed the level of ETS-1 activating phosphorylation by a specific anti-P-T38-ETS-1 antibody. WB analysis (Figure 2A) showed a significant inverse correlation between total ETS-1 and its T38-phosphorylated fraction during melanoma progression. As shown (Figure 2A and Supporting Information Figure S1) melanocytes and primary melanomas, expressing high levels of total ETS-1, displayed a barely or undetectable phosphorylated fraction, whereas most of the ETS-1 protein was activated through T38-phosphorylation in metastatic melanomas. These results correlated with the different phosphorylation status of ERK1/2. Considering the possible functional relevance of ETS-1 cellular topographic distribution, equal amounts of cytoplasmic and nuclear extracts obtained from Me1007 and A375M, as representatives of primary and advanced melanoma cell lines, were analyzed by immunoblot. Despite a significant amount of ETS-1 in the cytoplasm of both primary and advanced tumor cells (Figure 2B), the T38-phosphorylated fraction in the nuclear extracts was clearly visible only in the A375M metastatic cell line (Figure 2B).

Confocal immunofluorescence analysis confirmed the different level of P-T38-ETS-1 in primary versus advanced melanomas. In Me1007 primary melanoma cell line, the low level of nuclear P-T38 ETS-1 (red) made it difficult to detect when merged with the strongly stained total ETS-1 (green) (Figure 2C, left). Conversely, in the metastatic A375M cell line, a nuclear bright and scattered signal of P-T38 ETS-1 (red) colocalized in the merging picture (orange spots) with the slight nuclear portion of total ETS-1 (green). As evident in these metastatic cells most of the unphosphorylated ETS-1 is contained in the cytoplasm (Figure 2C, right).

Conversely, the considerable level of phosphorylation of serines at exon VII did not show any clear modulation among melanomas at different stages of disease (not shown).

### ETS-1 level is downregulated by miR-222

**Figure 3:**
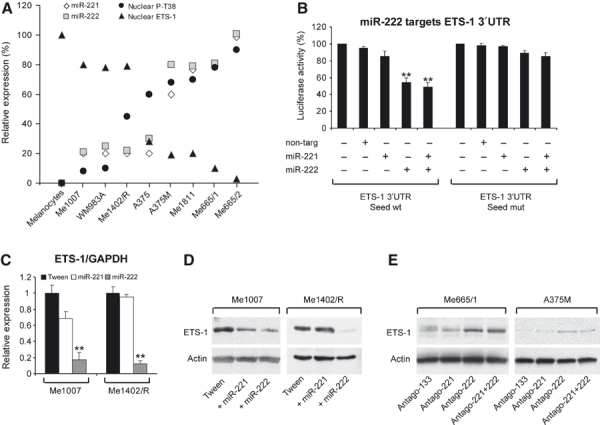
ETS-1 regulation by miR-221 and/or -222. (A) miR-221 and -222 expressions are inversely or directly correlated with ETS-1 or its P-T38 fraction in melanoma progression. Data were obtained by densitometric analysis of representative Western and Northern blots (Figure 1, 2A, S1 and not shown); in each curve the highest value is reported as 100%. (B) Luciferase (LUC) reporter assay (columns of a minimum of 3 experiments, bars ± SD) performed by cotransfecting miR-221 and/or miR-222 in the presence of the LUC reporter gene linked to ETS-1 3′UTR. As controls, mutated 3′UTR sequences and a non-targeting oligomer were also included. **P < 0.01. Evaluation by qRT-PCR (C) and WB (D) of ETS-1 expression in Me1007 and Me1402/R melanomas transduced with miR-221, miR-222 or control Tween vector. (E) WB analysis of ETS-1 in antagomir-treated A375M and Me665/1 metastatic cell lines. Antagomir-133 represents an irrelevant negative control. miR-221/-222 and antagomir oligos were utilized at 150 and 200 nM, respectively.

Based on the inverse correlation between ETS-1 and miR-221/-222 expression patterns (Figure 3A) and on bioinformatics analyses (TargetScan, RNAHybrid), predicting the presence of one conserved binding site in the 3′ UTR of ETS-1, we tested, using a luciferase assay, whether miR-221/-222 were able to directly target ETS-1. The ETS-1 3′UTR, encompassing the seed sequence theoretically recognized by miR-221 and -222, was cloned downstream to the luciferase open reading frame in a modified pGL3 promoter vector. Cotransfection experiments were performed in the 293FT cell line. We avoided the use of the whole 3.6 kb 3′UTR in the transfection assays in order to exclude a series of adenylate- and uridylate-rich repeats which may be involved in mRNA stability and translational regulation (Chen and Shyu, 1995). As shown in Figure 3B, transfections of miR-222 in the presence of ETS-1 3′UTR induced a significant decrease of the luciferase activity (roughly 50% less than non-targeted control sequence). Surprisingly, the effect induced by cotransfection of miR-221 was negligible, suggesting that the coordinated function reported for miR-221/-222 in the regulation of p27Kip, c-KIT (Felicetti et al., 2008) and other target genes (Garofalo et al., 2009) can be uncoupled. The control, cotransfection of ETS-1 3′UTR with a non-targeting sequence as well as of ETS-1-mutated 3′UTR with miR-221/-222, did not show any repression in luciferase activity, confirming the specificity of such interactions (Figure 3B). ETS-1 downregulation was confirmed at both mRNA and protein levels in miR-222-transduced Me1007 and Me1402/R primary melanoma cell lines (Figure 3C,D). In agreement with the luciferase results, overexpression of miR-221 did not show significant effects on ETS-1 (Figure 3C,D). The reverse effect on ETS-1 regulation was confirmed in Me665/1 and A375M metastatic melanomas by miR222-specific antagomir transfection. Western blot analysis demonstrated the capacity of antagomir-222 to raise the ETS-1 level compared with either antagomir-221 or the non-relevant antagomir-133 (Figure 3E). These results indicate that miR-222 is a true regulator of ETS-1 expression.

### ETS-1 regulates miR-222 transcription

**Figure 4:**
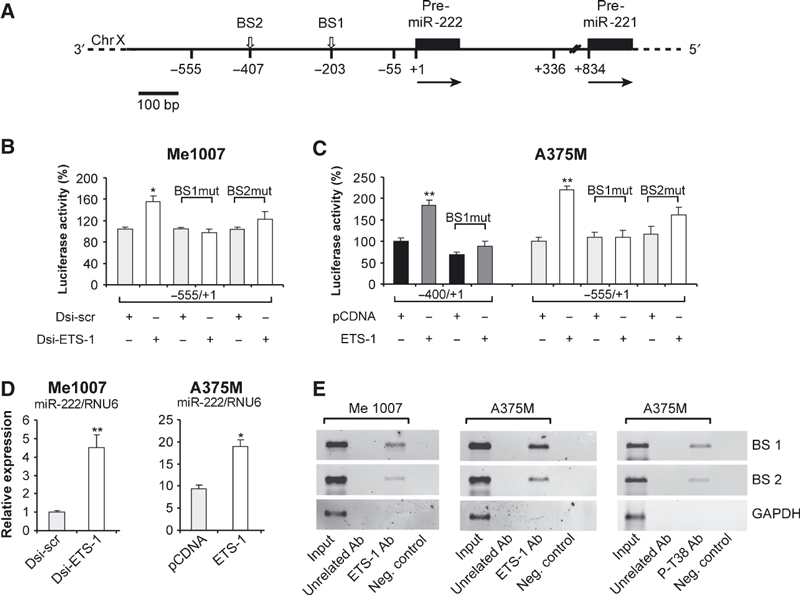
ETS-1 involvement in miR-221 and/or miR-222 transcriptional regulation. (A) Schematic depiction of the genomic region upstream of pre-miR-222. BS1 and BS2 indicate ETS-1 putative binding sites, and horizontal arrows the direction of microRNA transcription. (B) Promoter luciferase assays performed either in the presence of Dsi-ETS-1 (200nM) or ETS-1 in Me1007 primary and (C) A375M metastatic melanoma cell lines, respectively. (D) Evaluation of miR-222 levels by qRT-PCR in Dsi-ETS-1 or ETS-1-transfected cells compared with controls. (E) Chromatin immunoprecipitation assays in Me1007 (left) and A375M cells (middle and right) with anti-ETS-1 or anti-P-T38-ETS-1 antibodies (right).).*P < 0.05; **P < 0.01.

We reckoned that the increased level of P-T38 ETS-1 might be functional for growth and malignancy of cancer cells. Since we evidenced a direct relation between miR-221/-222 levels and the P-T38 form of ETS-1 in melanoma progression (see Figure 3A), we hypothesized that this regulation could be achieved through a miR222-ETS-1 loop, where miR-222 diminished the ETS-1 level, but the residual P-T38 ETS-1-activated fraction was in turn able to induce miR-222 transcription. To prove this hypothesis, we looked for ETS-1 binding sites (EBS) in the ∼0.6 kb sequence upstream to pre-miR-222/-221 (MatInspector software –http://www.genomatix.de) (Figure 4A). To test whether such putative EBS were truly functional, we performed a series of promoter luciferase assays. The sequence from −555 to +1 nt (containing −203 and −407 ETS-1 BS, named BS1 and BS2, respectively), as well as the shorter fragment from −400 to +1 nt (only containing −203 ETS-1 BS1) were cloned in a promoter-less pGL vector and cotransfected with empty or ETS-1-containing vectors at first in the highly transfectable 293FT cells (Figure S2) and then in Me1007 and A375M, as representative primary and metastatic melanoma cell lines (see Figure 4B).

A 30–45% ETS-1-dependent reduction of the luciferase activity was observed in the 293FT cells, likely due to ETS-1 binding to the miR-222 regulatory sequences. The introduction of point mutations in each core-binding site restored the luciferase levels totally or partially when mutations were in the BS1 (−203) or BS2 (−407) sites, respectively (Supporting Information Figure S2A). This suggests a different role/hierarchy for these two binding sites and/or the presence of interacting activators in the −555/−400 region. The longer −555/+1 fragment seems to induce a transcriptional activity higher than the shorter −400/+1 fragment (not shown).

To close the loop, ETS-1 would be expected to regulate miR-222 and perhaps miR-221. To test this, 293FT cells were transiently transfected with the ETS-1-expressing vector (Figure S2B, left) and the level of both endogenous miR-221 and -222 was evaluated by qReal-time PCR at early as well as late time points (from 4 to 48 h) (Figure S2B, right). A significant stepwise decrease of miR-222 followed ETS-1 transfection. In contrast, no clear modulation of miR-221 was observed, confirming the fidelity of the miR-222-ETS-1 liaison (Figure S2B, right). Western blot confirmed the lack of ETS-1 and ERK 1/2 phosphorylation (Figure S2C).

A similar ETS-1-dependent repressive function was observed when early stage Me1007 melanoma, expressing high levels of barely or unphosphorylated ETS-1, was used as a recipient for the promoter luciferase assays. Dsi-ETS-1 cotransfection, in the presence of the long (−555/+1) miR-222 promoter fragment, confirmed the ETS-1 negative regulation on miR-222 transcription (Figure 4B). In view of its lower activity we did not include the shorter promoter fragment (−400/+1). Notably, when the above cotransfection experiments were run in the A375M metastatic melanoma cell line, a substantially different picture was seen with ETS-1-inducing miR-222 promoter-driven luciferase (Figure 4C). A twofold induction was obtained by transfecting either the short or the long fragments containing one (BS1) or two (BS1 and BS2) ETS-1 binding sites, respectively. This induction was totally abrogated when the BS1 site was mutated either in the long or the short fragments of miR promoter. In contrast, BS2 disruption only partially restored the luciferase level (Figure 4C). According to these results, qRealtime PCR showed that miR-222 upregulation was a consequence of either ETS-1 silencing in primary melanomas or ETS-1-enforced expression in metastatic melanomas (Figure 4D). To verify direct binding of ETS-1 to BS1 and BS2 on miR-221/-222 promoter, we carried out chromatin immunoprecipitation (ChIP) assays in Me1007 and A375M melanomas (Figure 4E). ChIP assays of both cell lines showed a significant ETS-1 binding at the analyzed sites (BS1 and BS2), whereas no chromatin enrichment was observed in control reactions immunoprecipitated with an irrelevant antibody. Moreover, in the A375M metastatic cells we also demonstrated the binding competence of the Thr38 phosphorylated fraction of ETS-1 (Figure 4E, right). The above results support the view that ETS-1 has different activities on miR-222 promoter depending on cell context and, ultimately, its different phosphorylated status, as exemplified by the comparison between primary and advanced melanomas (Figure 4B,C).

**Figure 5:**
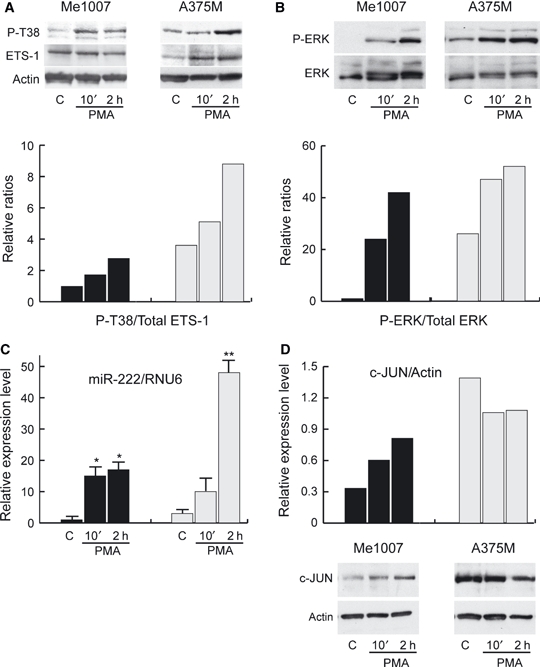
ETS-1 regulation in PMA-treated melanoma cells. (A) Top, representative WB analysis of P-T38 and total ETS-1 expression in Me1007- and A375M-treated cell lines as a paradigm of primary and metastatic melanomas; bottom, relative expression ratios, derived from densitometric analysis, shown by graph bars. (B) Top, representative WB analysis and relative densitometric values of P-ERK and total ERK (bottom). (C) qRT-PCR of miR-222 in the same PMA-treated samples. RNU6 was used for normalization. Values are mean ± SD of three independent experiments. *P < 0.05; **P < 0.001. (D) Representative WB analysis and relative quantification of c-JUN.

To verify the hypothesis of ETS-1 functional shift, in connection with P-T38 constitutive activation, we treated the primary Me1007 and the metastatic A375M melanoma cell lines with the phorbol ester PMA, which induces ETS-1 phosphorylation by stimulating ERK1/2 through the RAF/MEK signaling pathway (Jørgensen et al., 2005). Although at different levels and with different kinetics, both PMA-treated cell lines showed increased T38-ETS-1 phosphorylation coupled with P-ERK expression levels (Figure 5A,B). In Me1007 cells displaying low endogenous levels of P-T38, PMA induced a boost of phosphorylation as early as 10 min after treatment, whereas in A375M cells, which already displayed a high level of the phosphorylated fraction, a significant rise of P-T38 was observed only after 2 h. Of note, unlike A375M, the Me1007 genome does not carry any of the frequent mutations characterizing melanomas, such as point mutations in N-RAS or B-RAF genes. The levels of P-ERK and P-T38-ETS-1 were always higher in A375M metastatic than in Me1007 primary melanomas (Figure 5A,B). The P-T38/total ETS-1 ratio might tune the ETS-1/miR-222 loop in melanoma, a hypothesis that, beside promoter luciferase results, appears to rely on the concordant increment of P-T38-ETS-1 and miR-222 in PMA-treated Me1007 and A375M melanomas (Figure 5A,B) and on the direct correlation between the endogenously produced P-T38-ETS-1 and miR-222 in melanoma progression. The well known interplay already described between ETS-1 and c-JUN also appears to be functional to miR-222 regulation. In metastatic melanoma, the P-T38-activated fraction of c-ETS-1, by competing with c-ETS-1 for binding to DNA, may increase AP-1-driven transcription of those targets, such as miR-222, containing ETS-1 and AP-1 binding sites in close proximity on their promoters. Moreover, as reported (Goldberg et al., 1994) and confirmed by our small interfering experiments showing c-JUN upregulation in Dsi-ETS-1-transfected melanoma cells (results not shown), c-ETS-1 is able to repress c-JUN, interfering with its strong transcriptional regulation. Indeed, miR-222 is barely detectable in the presence of a low P-T38/total ETS-1 ratio and a low c-JUN level, and vice versa (Figures 3A and 5A,B,D).

### Functional role of ETS-1

To assess the functional role of ETS-1 in miR-222 regulation and disease progression, we tested the tumorigenic properties of melanoma cells forced either to overexpress or repress ETS-1. The Me665/2 metastatic melanoma, displaying a very low level of endogenous ETS-1, was transduced with the Tween-lentiviral vector overexpressing ETS-1. Although qReal time RT-PCR and Western blot analyses confirmed the correct transcription and translation of the transduced ETS-1 cDNA, no significant increase of nuclear ETS-1 protein was induced, most of the ectopically expressed ETS-1 being delocalized in the cytoplasm (Supporting Information Figure S3A). This result suggests that the nuclear shift of ETS-1 is highly regulated in malignant melanoma cells.

**Figure 6:**
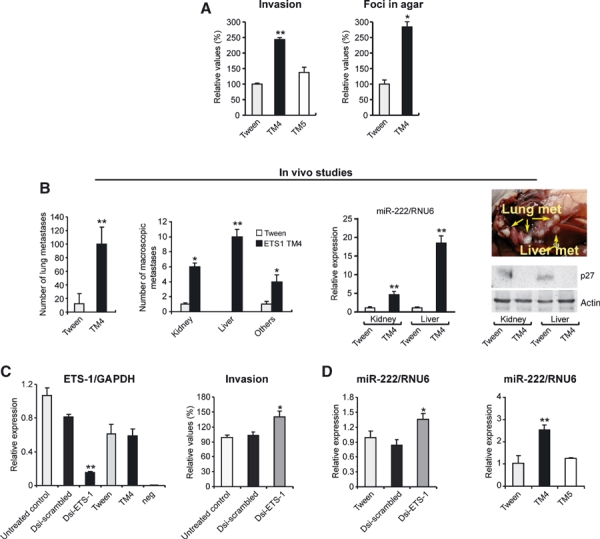
ETS-1 functional role in A375M metastatic melanoma. (A) Invasion assay (left), and evaluation of the number of foci in semisolid medium (right) in TM (dominant negative)-expressing cells versus controls. (B) In vivo studies in i.v. injected athymic nude mice evaluated as number of lung (left) and abdominal (middle) metastases; miR-222 and p27 expression were also evaluated in liver and kidney TM-derived metastases compared with controls; lung and liver metastases are indicated by arrows (right). Data, shown as median ± SD, were obtained from at least three independent experiments. (C) Left, qRT-PCR evaluation of ETS-1; right, invasion assay in A375M Dsi-ETS-1-transfected cells. (D) qRT-PCR of miR-222 in Dsi-ETS-1-transfected (left) and TM-infected A375M cells (right). RNU6 was used for normalization. *P < 0.05; **P < 0.01.

Clearer, although complex, results were obtained in ETS-1 competition/repression experiments by using a stable trans-dominant negative form (TM) or a transient specific Dsi-ETS-1. The TM-truncated form of ETS-1, containing only its binding domain, has been shown previously to compete with the wild-type protein for the DNA binding sites and the underlying functions (Figure S3B, left) (Nakano et al., 2000; Pourtier-Manzanedo et al., 2003). This truncated form of ETS-1 was mainly localized in the nucleus (Figure S3A). Six separate gene transductions (TM1–TM6) were performed in the metastatic melanoma A375M, and the A375M/TM4 melanoma cell line, selected for the highest amount of the truncated protein, was successively utilized in most of the functional analyses (Figure S3B, right). In vitro biological assays showed that TM-transduced cells proliferated slightly faster (20–30% increase) than the empty vector-transduced counterpart (not shown) and displayed a 2.5-fold increase of invasive capacity, as evaluated using a Boyden chamber assay (Figure 6A, left). This invasive activity correlated with the amount of TM expression, as shown by comparing A375M/TM4 and/TM5, with respectively high and low expression of the dominant negative protein (Figure 6A, left). A significant enhancement (of approximately threefold) of melanoma capacities of forming foci in agar semisolid medium was also observed in A375M/TM4 cells compared with vector-transduced cells (Figure 6A, right). Similar results were obtained in Dsi-ETS-1-transfected Me665/1 metastatic cell line (Figure S4).

The behavior of control Tween- and TM4-transduced A375M cells was also tested in vivo following intravenous injection in natural killer (NK)- depleted athymic nude mice. A significant increase in the number of lung metastases was generated by intravenous (i.v.) injection of TM4- compared with Tween-transduced cells (Figure 6B, left). Moreover, we observed TM4-induced abdominal metastasis dissemination, mainly to liver and kidney (Figure 6B). As expected, TM4-derived metastases displayed a significant increase of miR-222 and a correlated decrease of p27Kip compared with controls (Figure 6B, right).

We then considered the possibility that this Ets-dominant negative protein interferes with other members of the ETS family, although we never observed any significant modulation of ETS-2, the closest member to ETS-1, in our melanoma samples (data not shown). Nonetheless we cannot rule out the contribution of ETS family proteins besides ETS-1 to melanoma functional properties. To confirm an ETS-1-specific contribution unambiguously, we silenced it in A375M melanoma cells using DsiRNA, obtaining a 75% downregulation of ETS-1 as evaluated by qRT-PCR (Figure 6C, left) and Western blot (Figure S3C). Although the effect of ETS-1 knockdown on cellular invasion (Figure 6C, right) was less than that of TM (Figure 6A, left), results clearly confirmed a role of ETS-1 among all the ETS family genes possibly targeted by the TM dominant negative. The abrogation of ETS-1 mRNA and, even more, the expression of ETS-TM dominant negative were paralleled by a significant increase of miR-222, suggesting that both the DsiETS-1 and the TM protein were able to unblock miR-222 regulatory region by abrogating or competing with the serine phosphorylated fraction or the amount of unphosphorylated ETS-1 (Figure 6D and Supporting Information Figure S4).

Finally, bearing in mind that our previous data showed that the absence of PLZF underlies melanoma progression by unblocking miR-221/-222 transcription, we evaluated the possibility of an ETS-1/PLZF integrated function. For this, we have specifically silenced ETS-1 in control and PLZF-transduced A375M cells to evaluate miR-222 expression levels and invasion capabilities. As shown (Figures 6C,D and Supporting Information Figure S5), we confirmed a Dsi-ETS-1-dependent increase of miR-222 paralleled by an enhanced invasion. The presence of PLZF reduces the invasion capacity of approximately 60% with respect to the control A375M cell line, whereas the simultaneous abrogation of ETS-1 in A375M/PLZF/Dsi-ETS-1 transfected cells decreases this PLZF-dependent inhibition thus suggesting that ETS-1 is necessary for maximal PLZF suppressive effects.

## Discussion

MicroRNAs (miR) are non-coding small sequences that regulate gene expression by binding to the 3′UTRs of their target mRNAs (Calin and Croce, 2006). Growing evidence indicates that miRs are abnormally expressed in all types of cancer, where they act as either oncogenes or tumor suppressors (Voorhoeve, 2010). In addition, microRNA signatures have appeared as a new important parameter which will hopefully allow, in the not too distant future, the selection of a truly tailored therapy based on predicted risks (Galasso et al., 2010). In particular, miR-221 and -222 are among the microRNAs most implicated in cancer on the basis of their upregulation in a number of solid tumors (Di Leva et al., 2010; Galardi et al., 2007; Gillies and Lorimer, 2007; Medina et al., 2008; le Sage et al., 2007). Croce and his group (2009) described an increase of miR-221 and -222 in NSCLC and HCC through c-JUN induction and PTEN and TIMP3 tumor-suppressor targeting (Garofalo et al., 2009). We have described miR-221 and -222 upregulation in melanomas and their targeted repression of p27Kip and c-KIT receptors, responsible for enhanced proliferation and blockage of differentiation, respectively (Felicetti et al., 2008). Here we have identified ETS-1 as another gene directly regulated by miR-222, but not by miR-221, demonstrating the existence of miR-221/-222-independent functionalities, in addition to the common ones, as recently reported for miR-222 in inflammation-mediated neovascularization through STAT5A (Dentelli et al., 2010). As reported (Filipowicz et al., 2008), the biological activities played exclusively by miR-221 or -222 should rely on the differences in the nucleotide sequence, apart of the seed, which can improve or reduce site efficacy. Despite sharing the same seed sequence (nt 2–8), nucleotides 13–16, which seem to play a significant role in microRNA function, are quite different in miR-221 and -222.

ETS-1 transcription factor is the founding member of the ETS gene superfamily, encoding a class of phosphoproteins characterized by a conserved domain that recognizes and binds to a GGAA/T DNA core sequence. ETS-1 is involved in an array of cellular functions, acting either as a positive or a negative regulator (He et al., 2007; Nakayama et al., 1999). The presence of ETS-1 in tissues derived from the neural crest, as melanocytes, might suggest the association of ETS-1 downregulation with the loss of the neural crest-associated phenotype during melanoma progression; nevertheless, our data speak in favor of its role in tumorigenesis. Of interest is also the similarity between ETS-1 and c-KIT. Like ETS-1, c-KIT is directly targeted by miR-221/-222 (Felicetti et al., 2008), being involved in normal cell growth and differentiation and repressed in metastatic melanomas (Alexeev and Yoon, 2006; Huang et al., 1998).

Although ETS-1 gene expression correlates with progression of tumors such as thyroid, pancreas, liver, lung and breast carcinomas (Seth and Watson, 2005), controversial data exist for melanoma (Rothhammer et al., 2004; Torlakovic et al., 2004).

Our results, showing miR-222-induced downregulation of ETS-1 during melanoma progression, at first sight speak against a possible tumorigenic role of ETS-1. To obtain a more realistic interpretation, we considered ETS-1 post-translational modifications, rather than its total protein content, functionally relevant to melanoma. In particular, a significant role should be played by the T38-phosphorylated fraction of ETS-1 and, more specifically, by the ratio of P-T38 and the total amount of ETS-1 protein, downstream to the RAS/RAF/MAPK pathway (Grenningloh et al., 2008; Pufall et al., 2005).

This ETS-1 complex regulation might possibly explain the controversial data describing ETS-1 as either directly involved in determining the malignant phenotype (Rothhammer et al., 2004) or a useless marker unable to distinguish between benign and malignant melanocytic lesions (Torlakovic et al., 2004). Torlakovic and collaborators (2004) analyzed a large collection of nevi, primary and metastatic melanomas from their archives for ETS-1 expression. They showed ETS-1 downregulation associated with tumor progression, with an inverse correlation between its expression and melanoma dimension, but no correlation with the tumor thicknesses. Further analyses of post-transcriptional and/or post-translational modifications, as well as of ETS-1 topographic cell distribution, might clarify this issue.

Indeed, in melanoma ETS-1 has a definitely more complex role than the apparent tumor-suppressive one, simply based on its downregulated expression in advanced tumors. In fact, melanoma malignancy is associated more with the RAS/RAF/ERK constitutive activation of ETS-1 than with ETS-1 total quantity, which also includes variable amounts of unphosphorylated or serine-phosphorylated fractions. Specifically, we have shown that primary melanomas display significant amounts of ETS-1, barely or not phosphorylated at T38, whereas metastatic cells show the reverse pattern, with low levels of ETS-1 highly phosphorylated at T38. This difference was particularly evident when nuclear proteins were compared (see Figure 2B,C). A similar regulation has been shown in Ki-RAS-transformed prostate cells where the activation of the RAF/MEK/ERK signaling pathway induces ETS-1 phosphorylation at T38, nuclear translocation and tumor progression (Cho et al., 2008; Mylona et al., 2006). In addition, the expression of miR-221 and miR-222 has been reported to be under the positive control of the RAS-MAPK signaling pathway in myoblasts induced to differentiate and in post-mitotic myotubes (Cardinali et al., 2009) and in PC12 cells (Terasawa et al., 2009). Our results might fill the gap between MAPK and miR-222, demonstrating P-T38-ETS-1 protein as an intermediate transcriptional regulator.

Considering that in non-small cell lung cancer and hepatocarcinoma cells c-JUN has been involved in miR-221/-222 activation (Garofalo et al., 2009), it is also important to point out the capability of ETS-1 to associate and cooperatively bind with the AP-1 transcription complex to regulate gene expression (Chang et al., 2003). Our data confirm that, in metastatic melanomas, the ERK constitutive signaling activates c-JUN (Lopez-Bergami et al., 2007) and P-T38-ETS-1 (Yang et al., 1996), which in turn seem to cooperate towards miR-221/-222 activation (Figure 5) (Garofalo et al., 2009). Of note, the increment of miR-222 observed in the A375M metastatic melanoma after a 2-h PMA treatment was coupled with a simultaneous activation of P-T38-ETS-1, whereas the constitutive high expression of c-JUN appeared unmodified, thus suggesting that a higher P-T38/ETS-1 ratio is an essential step for miR-222 regulation and, consequently, melanoma malignancy (Figure 5). Finally, it is important to mention the intriguing results obtained by transducing the dominant negative TM ETS-1 in the A375M and Me665/1 melanomas in that it induces a more aggressive behavior with respect to control cells both in vitro and in vivo. The increased levels of miR-222 and, in turn, of tumorigenesis induced by the dominant negative truncated form of ETS-1 is probably derived from the competitive displacement of the inhibitory components of this protein. Considering that opposite functions of ETS-1 have been already described and linked to the different biological contexts (Sato et al., 2001), it is likely that in melanoma cells the net establishment of a more aggressive phenotype could derive from undermining the nuclear unphosphorylated and, even more, the serine-phosphorylated ETS-1 fractions characterized by a significantly lower DNA affinity (Pufall et al., 2005). In this picture, the TM-dependent invasive potential of the A375M cells in nude mice (a more than fivefold increase in the number of lung metastases and dissemination to additional areas) seems, at least in part, the consequence of the enhanced miR-222 expression coupled with targeted repression of tumor suppressors such as p27Kip (Figure 6). Nonetheless, TM ETS-1 might also compete with other ETS-1-regulated tumor suppressor genes, such as DUSP6 in lung cancer (Zhang et al., 2010).

Taken together, our data suggest a complex ETS-1/miR-222 coregulatory loop where the balance between miR-222 and this target is differentially modulated in normal and neoplastic cells. Indeed, ETS-1 loses its normal repressive function in melanoma, becoming a positive regulator of miR-222. This is a consequence of MAPK constitutive activation leading to high levels of P-T38, the ETS-1-activated form that seems to cooperatively act with PLZF and c-JUN as fine-tuners of miR-222. In this context, high c-JUN expression should be considered a necessary, but not sufficient, step. Moreover, ETS-1 expression seems to be required for effective PLZF repression (Figure S5). Conversely, P-Ser and unphosphorylated ETS-1 molecules should downregulate c-JUN (results not shown). Finally, it is important to consider that such a complex regulation, being just a part of the numerous molecular pathways involving ETS-1, underscores the importance of further analyses absolutely required before any possible translation to therapy. These data represent another piece of evidence substantiating the inhibition of miR-221 and -222 as a promising approach for translation to the clinical setting by restoring the underlying signaling pathways.

## Methods

### Cell lines culture and transduction

Most of the human melanoma cell lines used in the current study was stabilized from surgical specimens obtained from primary or metastatic tumors at Istituto Nazionale Tumori in Milan (Italy). Cell lines were characterized for growth in soft agar and, whenever possible, their metastatic potential was evaluated in athymic nude mice. Stages and references of these cell lines are listed in Carè et al. (1996). The A375 cell line was from the American Type Tissue Collection (Rockville, MD) and its metastatic variant A375M was kindly provided by Dr. R. Giavazzi (Istituto M. Negri, Bergamo). Normal human epidermal melanocytes from foreskin were obtained from Promocell (Heidelberg, Germany).

To modulate ERK1/2 activity, melanoma cells were treated with 50 nM of phorbol-12-myristate-13-acetate (PMA) at 37°C up to 2 h (Jørgensen et al., 2005).

The ETS-1 cDNA, encompassing its complete coding sequence, and the ETS-1 transdominant negative mutant TM were cloned in the retroviral Pinco and the lentiviral Tween vectors, respectively (Lulli et al., 2010). Overexpression of PLZF and miR-221/-222 were obtained in melanoma cells by using retroviral and lentiviral vector systems, as reported (Felicetti et al., 2008). ‘Controls’ are always designed as empty vector-transduced cell lines.

ETS-1 was specifically silenced using small interfering RNA (IDT, Leuven, Belgium). Briefly, 24 h after plating, cells were transfected using Lipofectamine 2000 (Invitrogen srl, San Giuliano Milanese, MI, Italia) with either DsiETS-1 (HSC.RNAI.N001143820.10.1) or a DsiRNA scrambled control (final concentration 200 nM). The level of ETS-1 mRNA was analyzed 48 h after transfection by qReal-time PCR (Applied Biosystems assay #Hs00428287, Life Technologies Corporation, Carlsbad, CA, USA) and the functional properties of the cells evaluated up to day 5.

### MicroRNA-221 and -222 silencing by antagomir treatment

Chemically modified antisense oligonucleotides (antagomir) were used to inhibit miR expression in vitro and in vivo. The sequences of antagomir-221 and antagomir-222 used were 5′P-GAAACCCAGCAGACAAUGUAGCU-3′-Chl and 5′P-GAGACCCAGUAGCCAGAUGUAGCU-3′-Chl, respectively; all the bases were 2′-OMe-modified. Antagomir oligonucleotides (Dharmacon Inc., Lafayette, CO, USA), were transfected at doses ranging from 50 to 200 nM using Fugene HD (Roche Diagnostics Spa, Monza, MI, Italia), according to the manufacturer's procedures. As controls, an unrelated antagomir (specifically the antagomir targeting miR-133a that is not expressed in melanomas) and an FITC-conjugated oligo targeting the luciferase sequence (ratio FITC oligo:antagomir 1:10) were transfected as well. Transfection efficiencies were analyzed by FACS.

### Western blot

Western blot was performed according to standard procedures. Cell lysates were separated by the pre-cast NuPAGE polyacrylamide gel system (Invitrogen). Antibodies against ETS-1 (anti-rabbit from Santa Cruz Biotechnology, Santa Cruz, CA, USA and anti-mouse from Novocastra, Newcastle, UK), ETS-2 (Santa Cruz Biotechnology), ETS-1-P-T38 and ETS-1-P-S282/285 (Biosource International Inc., Camarillo, CA, USA), c- JUN (Santa Cruz Biotechnology) ERK1/2, P-ERK1/2 and p27Kip1 (Cell Signaling Technology Danvers, MA, USA) were used in accordance with the manufacturer's instructions. Actin (Oncogene Research, San Diego, CA, USA), tubulin (Sigma, St. Louis, MO, USA) and nucleolin (Santa Cruz Biotechnology) were used as controls for loading and specificity. The expression levels were evaluated by Scion Image Software (http://www.scioncorp.com).

### Promoter luciferase assay

To analyze the functional roles of the two putative ETS binding sites (indicated as BS1 and BS2 in Figure 4), a DNA fragment containing the putative regulatory region upstream to miR-222/-221 (from −555 to +1 nt) was amplified and cloned in pGL3 basic (Promega, Madison, WI, USA). A shorter construct (from −400 to +1 nt) containing the sole BS1 was also prepared. 293FT, Me1007 or A375M cell lines were transfected with Lipofectamine 2000 (Invitrogen) and (i) 200 ng of pGL3 basic or pGL3 containing the above genomic fragments, (ii) 150 ng of empty or ETS-1-expressing plasmid, and (iii) 30 ng of Renilla. At 48 h, cells were lysed and their luciferase activity measured using the FemtomasterFB 12 (Zylux, Huntsville, AL, USA). The wt pGL3 plasmids cotransfected with the control vector were considered to be 100%. As controls of specificity, point mutations were inserted in the wild-type core-binding sequence for ETS-1 using the QuickChange site-directed mutagenesis kit (Agilent Technologies, Santa Clara, CA, USA). Bold letters indicate the core consensus sequence. Mutated nucleotides are shown in lower case letters.

BS1: 5′-TGCTGC**gaGc**TCTCCAG -3′; BS2: 5′-TGACt**GcAg**GCAACA -3′.

### Chromatin immunoprecipitation (ChIP) assay

Five × 10^6^ cells from A375M melanoma cell line were fixed in 1% formaldehyde for 10 min at room temperature. Cells were washed with ice cold 1× PBS, scraped in 1× PBS plus protease inhibitors and collected by centrifugation. Cell pellets, resuspended in cell lysis buffer (50 mM Tris–HCl pH 8.0, 10 mM EDTA, 1% SDS) in the presence of protease inhibitors, were sonicated. DNA-protein complexes were immunoprecipitated using 3 μg of anti-ETS-1, anti-P-T38 or, as an internal control, the unrelated anti-DVL-1 (Santa Cruz). DNA-protein crosslinks were reversed by heating at 65°C overnight. The recovered DNAs were then PCR-amplified with the following primer set: DIR 5′-GGATCTACACTGGCTACTGAG-3′ and REV5′-GTCACAAGGAATCATGTATGC-3′, corresponding to ETS- binding site at -203 (BS1); DIR5′-CAGCATACATGATTCCTTGTGA-3′ and REV 5′-CTTTGGTGTTTGAGATGTTTGG-3′, ETS binding site at –407 (BS2). Control amplification was carried out on input chromatin (preserved before immunoprecipitation) and on DVL-1 (mock)-immunoprecipitated chromatin. To confirm the specificity of the immunoprecipitated products, GAPDH PCRs were also run.

### In vivo assay

For the in vivo assays, empty vector- or TM-transduced A375M cells in exponential growth phase were injected subcutaneously (s.c.) and i.v. at the dose of 10^6^ cells in adult athymic nude mice, minimum n = 5 mice per group (Charles River, Calco, Italy) pretreated with rat anti-mouse mAb to interleukin (IL)-2Rb (TMb1, the kind gift of Daniela Mannel, Regensburg, Germany) to deplete NK cells. Tumor growths were monitored twice a week. Six weeks after the injection, mice were sacrificed for necropsy and macroscopic metastases evaluation. Lung metastases were counted under a stereomicroscope after staining with India ink. Mice were treated and maintained at the Istituto Nazionale Tumori (Milan, Italy) according to institutional guidelines.

### Statistical analysis

Unless otherwise stated, results were representative of at least three independent experiments. All data were presented as mean values ± standard deviation (SD) or standard errors (SE). Statistical analysis was performed using the *t*-test with P > 0.05 deemed statistically significant. See also Appendix S1 for supplementary methods.

## Supporting information

**Figure S1.** Representative western blot of ETS-1 and its P-T38 phosphorylated fraction in normal human melanocytes (NHEM) and metastatic melanoma cell lines.

**Figure S2.** A, Promoter luciferase assays obtained by cotransfecting different genomic fragments (as indicated in Figure 4A) in 293FT cells in presence or not of ETS-1. As controls, mutations of core nucleotides have been included. Data are representative of at least three independent experiments. *P < 0.05. B, qRT-PCR analysis at different time points of ETS-1 mRNA (left) and miR-221/-222 (right). C, WB of ETS-1 and its P-T38-fraction, ERK1/2 and P-ERK1/2 in ETS-1 transfected 293FT cells. A375M is included as a positive control.

**Figure S3.** A, western blot analysis of ETS-1 and P-T38-ETS-1 in stably infected Me665/2/ETS-1 and A375M/TM4 melanoma cell lines compared with the corresponding empty vector transduced cells. Nuclear and cytoplasmic extracts have been analyzed. Actin is shown as a loading control. B, left, schematic picture of ETS-1 protein and its dominant negative (TM) truncated form; right, WB analysis of TM-transduced-A375M (TM1 to TM6 represent six independent lentiviral infections). C, western blot analysis showing ETS-1 downregulation in Dsi-ETS-1 transfected A375M cells. Dsi-scrambled is included as a negative control and actin is shown as a loading control.

**Figure S4.** ETS-1 functional role in Me665/1 metastatic melanoma. qRT-PCR of ETS-1 and miR-222 in Dsi-ETS-1 transiently transfected Me665/1 cells (top). GAPDH and RNU6 were used to normalize. Invasion assay in the same Dsi-ETS-1 transfected cells (bottom). *P < 0.05; **P < 0.01.

**Figure S5.** ETS-1/PLZF integrated function. qRT-PCR of ETS-1 and miR-222 in Dsi-ETS-1 transiently transfected A375M/PLZF cells (top). Relative invasion capabilities of A375M control, A375M/PLZF and A375M/PLZF/ Dsi-ETS-1 cells (bottom). A Dsi-scrambled sequence was included as a negative control. *P < 0.05; **P < 0.01.

**Figure S6.** Schematic representation of ETS-1-miR-222 regulatory circuitry in primary and metastatic melanomas. In primary melanomas lacking significant genomic alterations, as Me1007, high levels of inactive not phosphorylated ETS-1 repress miR-222 transcription. Conversely, in metastatic cells, as A375M, the constitutive activation of ERK1/2 induces ETS-1 phosphorylation at Thr38 and activates miR-222, possibly in cooperation with c-JUN. In turn, high amounts of miR-222 downregulates ETS-1, sustaining a high P-T38/total ETS-1 ratio.
