# Supplementary figures and images for: Constitutive activation of the ETS-1-miR-222 circuitry in metastatic melanoma
Source: Pigment Cell Melanoma Res. 2011 Jun 28;24(5):953–65. doi: 10.1111/j.1755-148X.2011.00881.x (PMC3272348; doi:10.1111/j.1755-148X.2011.00881.x)

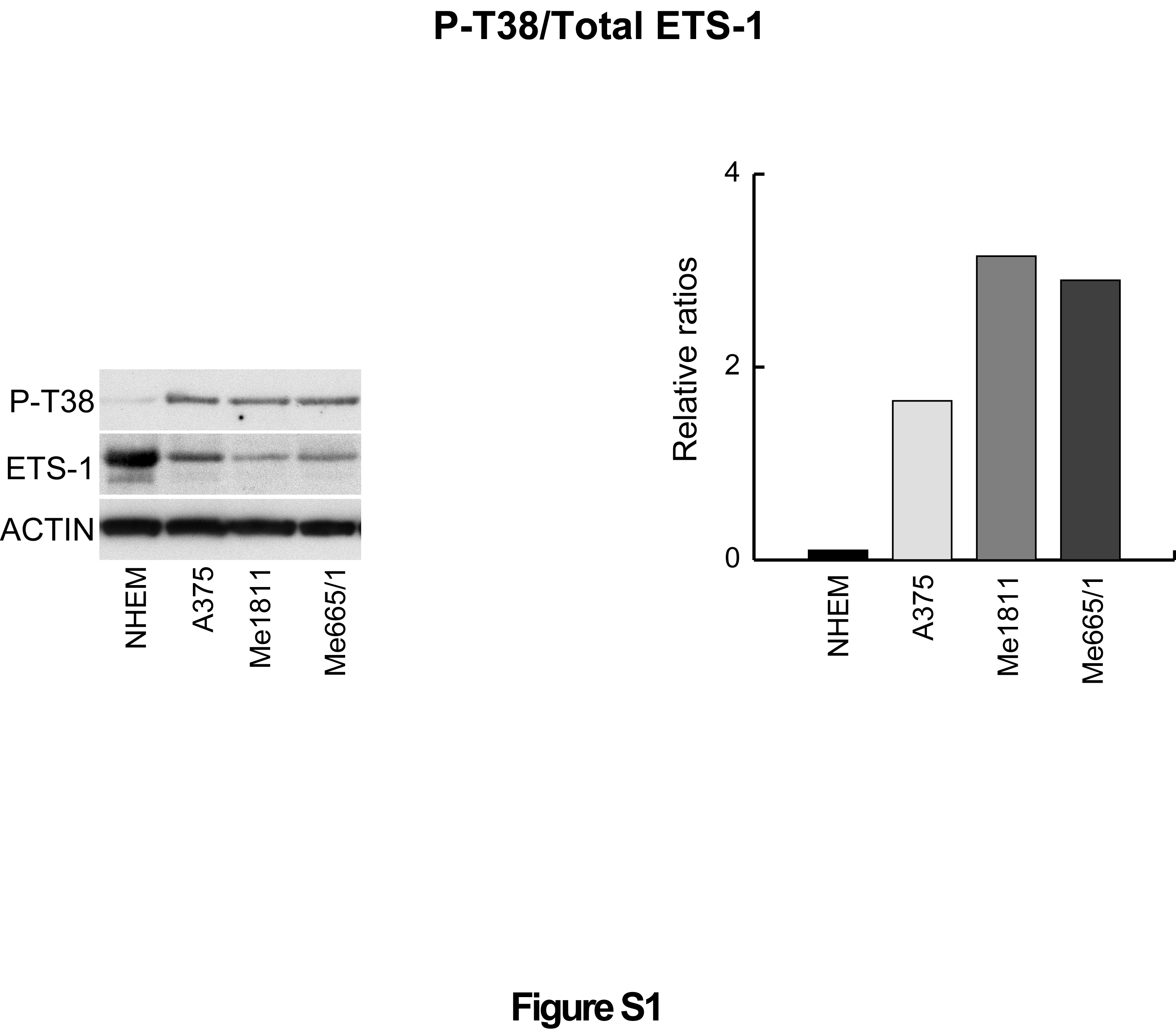

Supplement: Supplementary file 1 [file pcmr0024-0953-SD1.tif]

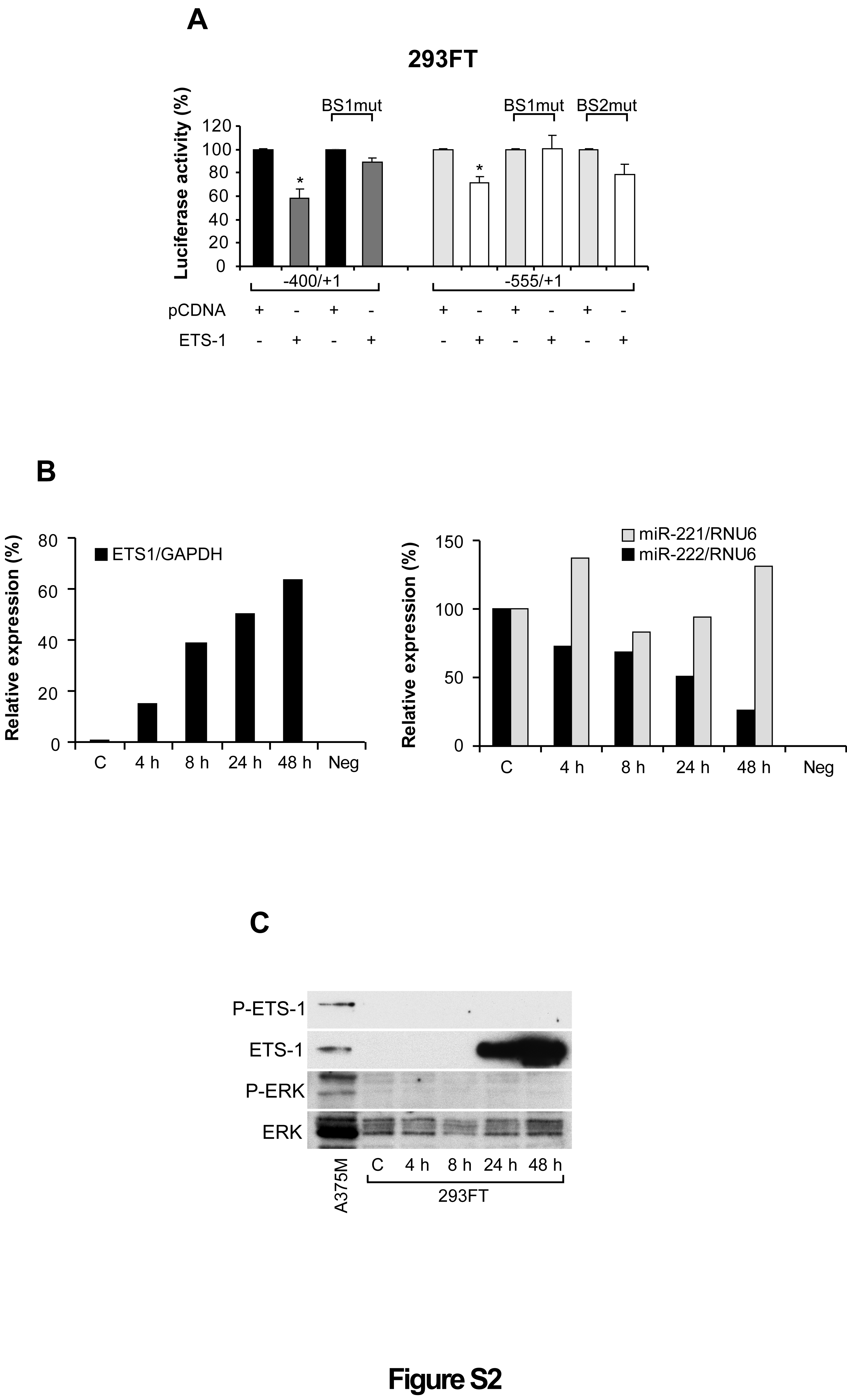

Supplement: Supplementary file 2 [file pcmr0024-0953-SD2.tif]

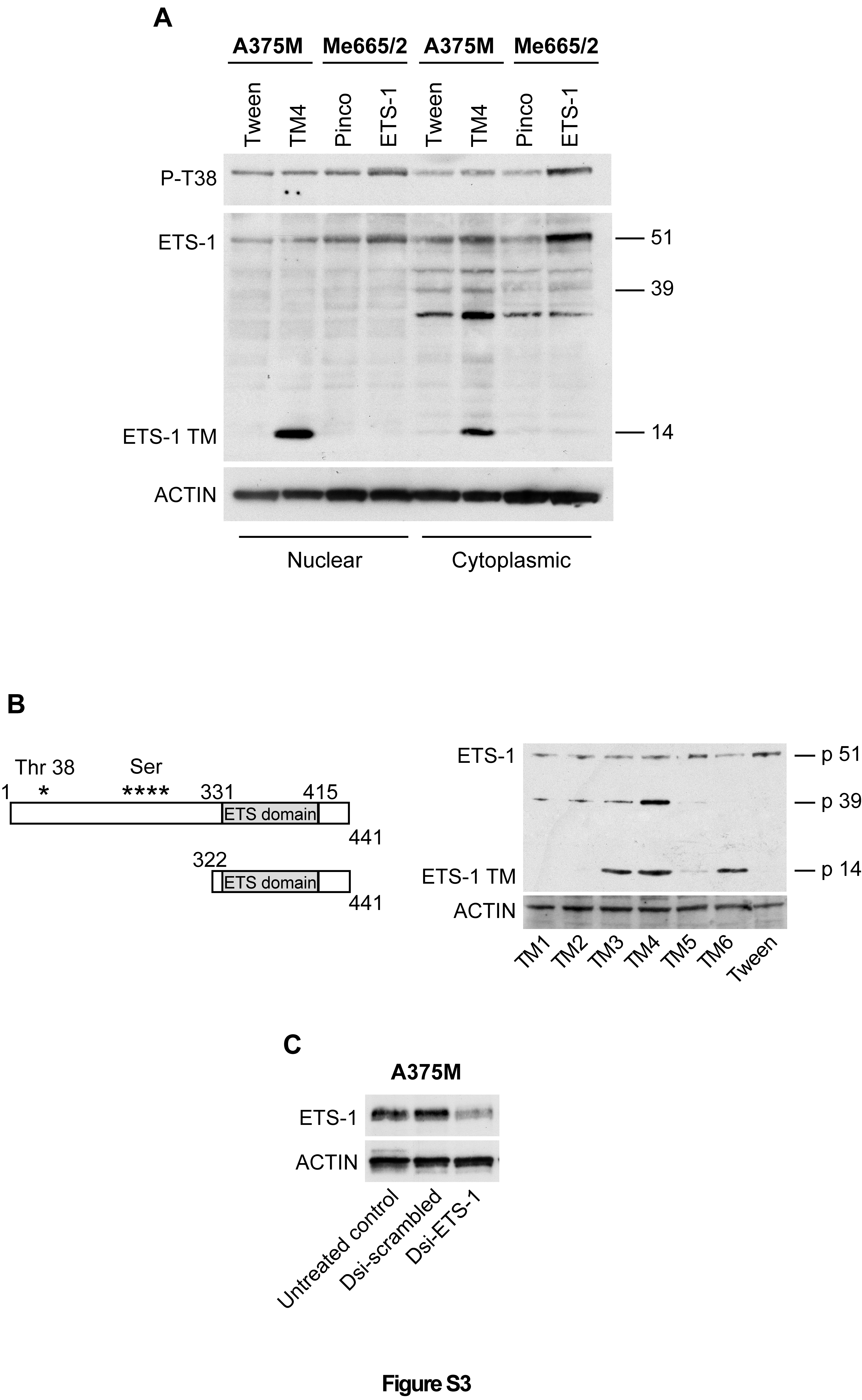

Supplement: Supplementary file 3 [file pcmr0024-0953-SD3.tif]

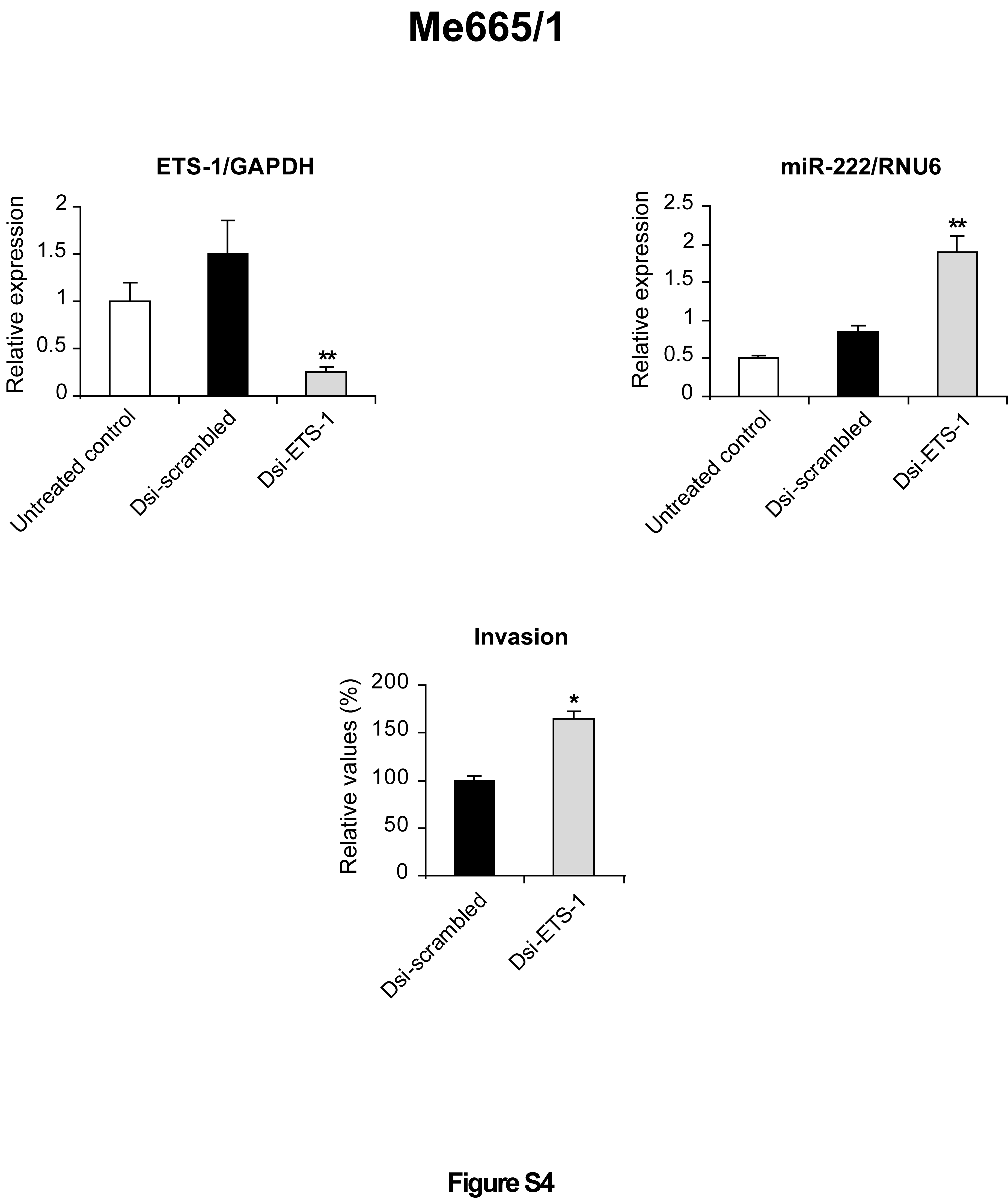

Supplement: Supplementary file 4 [file pcmr0024-0953-SD4.tif]

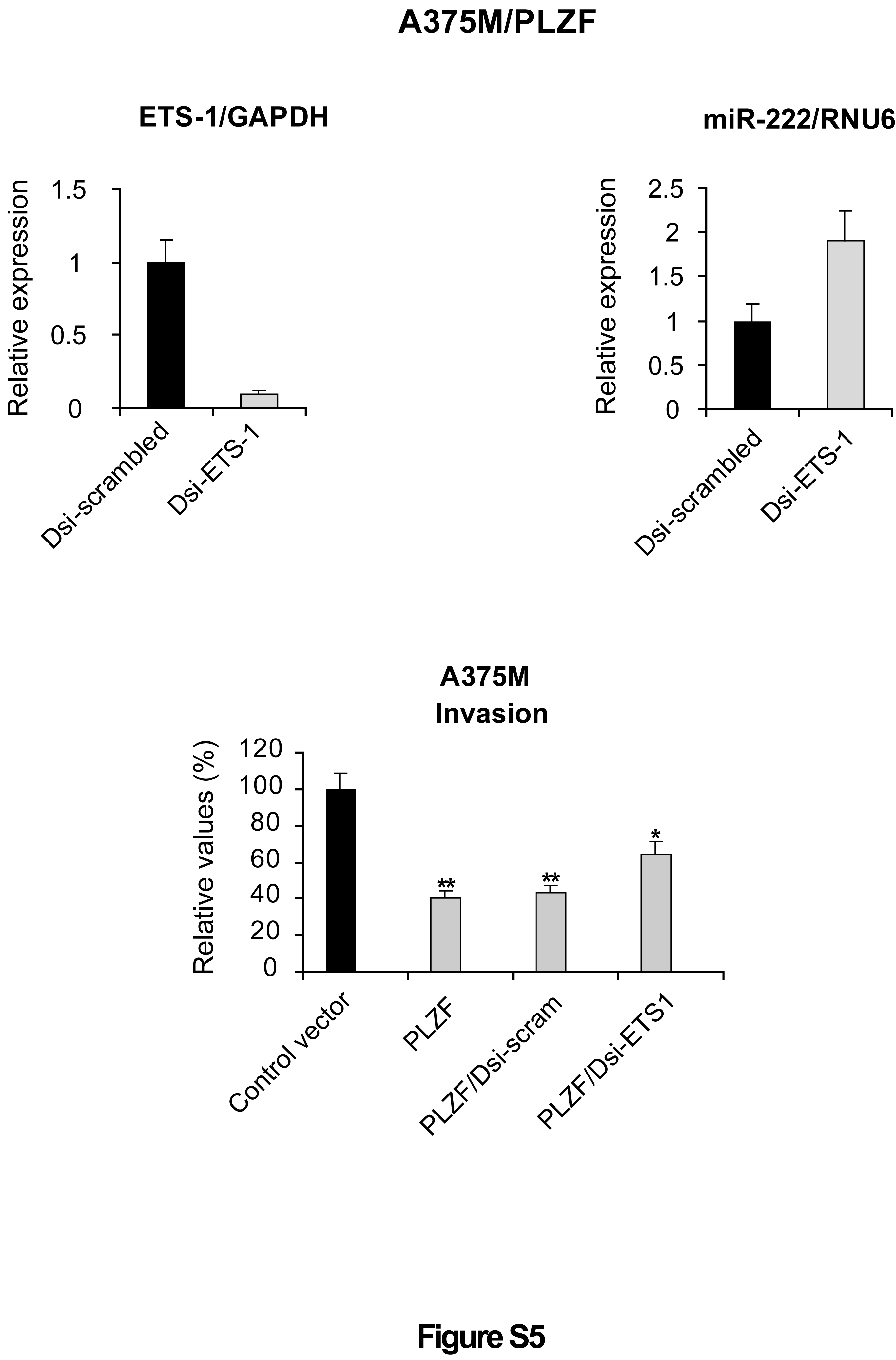

Supplement: Supplementary file 5 [file pcmr0024-0953-SD5.tif]

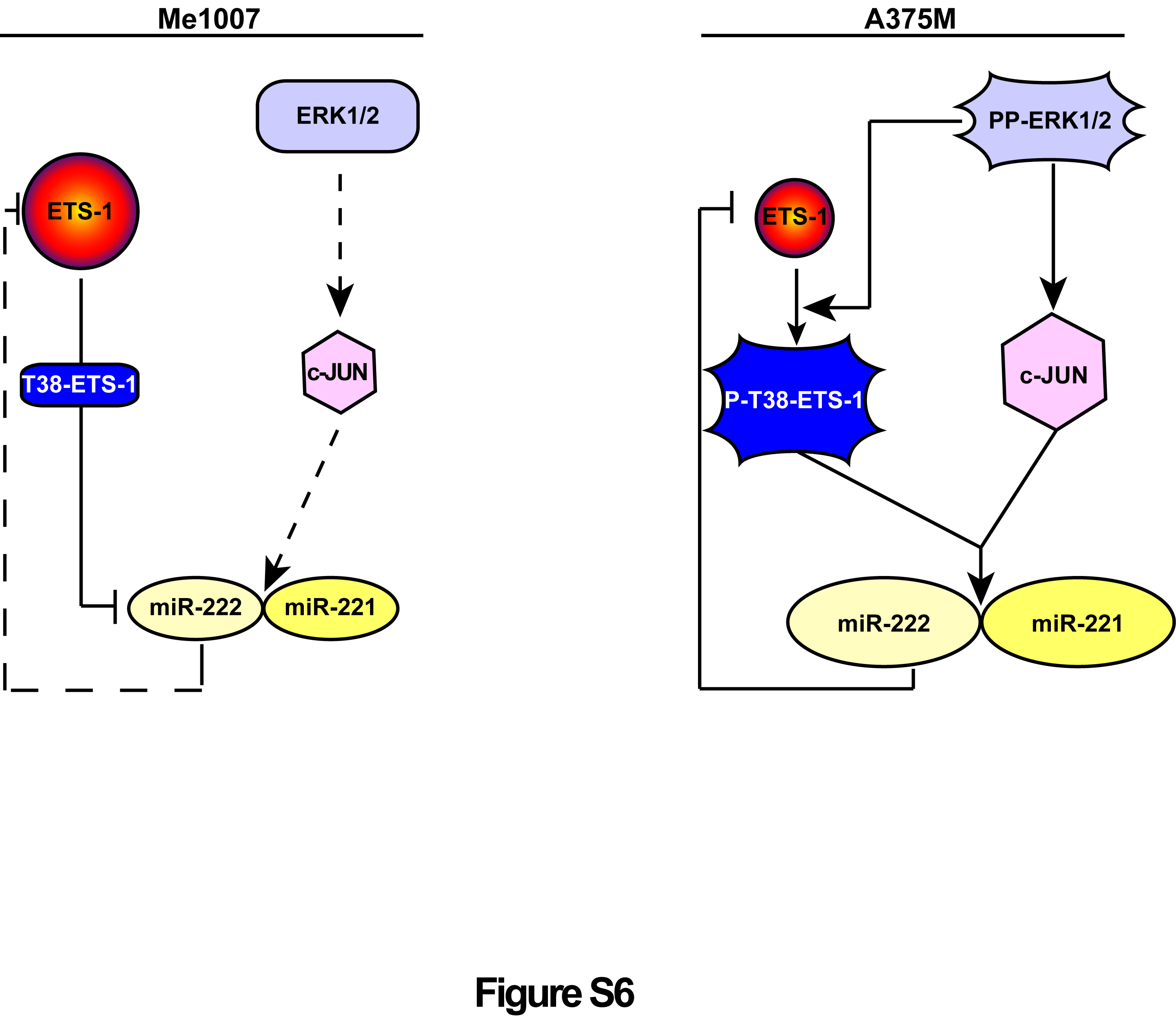

Supplement: Supplementary file 6 [file pcmr0024-0953-SD6.tif]
