# Supplementary material for: Constitutive activation of the ETS-1-miR-222 circuitry in metastatic melanoma
Source: Pigment Cell Melanoma Res. 2011 Jun 28;24(5):953–65. doi: 10.1111/j.1755-148X.2011.00881.x (PMC3272348; doi:10.1111/j.1755-148X.2011.00881.x)
Supplement: Supplementary file 7 [file pcmr0024-0953-SD7.doc]

**Supplementary Methods**

**Expression studies**

**Semiquantitative RT-PCR** was performed according to standard procedures. The sequences of primers and the annealing conditions were: ETS-1 dir 5’-ACTTACCCCTCGGTCATTCTCCG-3’ and rev 5’-CCACCTCATCTGGGTCAGAAAG-3’and the annealing temperature was 58° C. When required, the amplified fragments were hybridized to an internal 30 base end-labelled oligonucleotide according to Southern blot standard procedures.

**qRT-Real time PCR** Real time quantification method was performed according to the TaqMan MicroRNA Assays from Applied Biosystems (miR-221 #4373077; miR-222 #4373076). Samples were normalized by evaluating U6 expression (#4373381).

**Immunofluorescence assays** Me 1007 and A375M cells were growth in chamber slides up to semi-confluence, fixed in 4% paraformaldeide at room temperature (10 min.) and permeabilized in PBS containing 0.1% Triton, 1% BSA. The slides were blocked in 3%BSA/PBS for 1h. Primary antibody incubations were performed with 1:50 dilution for the mouse monoclonal anti-ETS-1 antibody (Novocastra, Newcastle, UK) in PBS containing 3%BSA, 0.05%Tween-20 at room temperature in humid chamber for 1 h followed by 1:40 dilution of the rabbit polyclonal anti-T38-Ets1 phosphospecific antibody (Biosource International Inc, Camarillo, CA, USA). For fluorescence staining an Alexafluor-488 anti-mouse and Alexafluor-555 anti-rabbit were used as secondary antibodies. After three washes, the slides were mounted with Prolong Gold antifade reagent and analyzed with confocal microscope (Olympus FV1000, Tokio, Japan) using fixed parameters for the two cell types.

**Target analysis**. Bioinformatic analysis was performed by using these specific programs:

TargetScan (<http://www.targetscan.org/>),PicTar (http://pictar.bio.nyu.edu/) and RNAhybrid (<http://bibiservice.techfak.uni-bielefeld.de/>).

**pGL-3’UTR plasmid.** For luciferase reporter experiments, on the basis of bioinformatic analyses, we identified one conserved putative binding site predicted to interact with miR-221 and -222 in the ETS-1 3’ UTR. This region was amplified by PCR (AccuPrime) from normal human genomic DNA using a Taq DNA polymerase high fidelity (Invitrogen). After sequence analysis, the construct was subcloned into the pGL3 promoter vector (Promega), immediately downstream from the stop codon of luciferase gene. The putative ETS-1 seed (nt 3403) and its mutated version were 5’TTAGAG**ATGTAGC**GATGTA 3’ and 5**’**TTAcAG**c TGccaC**GATGTA 3’, respectively. The conserved core is indicated in bold while lower case letters represent the mutated nucleotides. 293FT cells (5x10^4^ cells per well) were transfected with: (a) 20 ng of pGL3-3’ UTR plasmid, (b) 15 pmol of either a stability-enhanced 2’-O-Methyl non targeting RNA control or miR-221 and/or miR-222 oligonucleotides (Dharmacon Inc.), (c) Lipofectamine 2000 (Invitrogen) and (d) 50 ng of Renilla. At 48 h cells were lysed and their luciferase activity measured by using the FemtomasterFB 12 (Zylux). Ratios between Firefly and Renilla luciferase activities were measured with a dual luciferase assay (Promega). The wt pGL3-3’UTR cotransfected with the control non targeting oligonucleotide was considered as 100%.

**Functional assays.**

***In vitro* growth assay.** The proliferative rate of melanoma cells was evaluated by an XTT-based colorimetric assay (Roche Molecular Biochemicals). Cells, grown for different times in a 96-well tissue culture plate, were incubated with the XTT solution for 2 h. After this incubation period, orange formazan solution is formed, which is spectrophotometrically quantified using an ELISA plate reader (VICTOR2, Wallac).

***In vitro* invasion assay.** Invasion was assayed, as previously described (Felicetti et al., 2008), using cell culture inserts (Corning Costar Corporation) with 8μm pores coated with 100μg/cm^2^ of Matrigel growth factor reduced (Becton Dickinson) as a barrier. Cells (10^5^) were placed in the upper compartment in 100 μl of DMEM serum-free, while 600 μl of DMEM supplemented with 10% FBS were placed into the lower compartment of the chamber. Assays were incubated at 37° C in 5% CO2. After 24 or 48h the cells attached to the upper side of the membrane were removed with a cotton swab; each membrane was fixed and stained with crystal violet solution. Invasiveness was evaluated, as relative number of cells on the undersurface of the membrane, by a colorimetric assay at 595 nm in a microplate reader (VICTOR2, Wallac). The data were expressed as the mean absorbance ± SE for triplicate wells.

**Growth in semisolid medium.** Base layers of complete DME medium containing 0.5% agar were set in 60 mm plastic dishes. The bottom agar was overlaid with 1.5 ml of 0.33% agar containing suspensions ranging from 10^3^ to 10^4^ cells. Cultures were incubated for 3-4 weeks at 37°C and the colonies counted using an inverted microscope. Two experiments were performed for each cell line and results were calculated as the average ± SE of three dishes for each condition.
