# Supplementary material for: Constitutive activation of the ETS-1-miR-222 circuitry in metastatic melanoma
Source: Pigment Cell Melanoma Res. 2011 Jun 28;24(5):953–65. doi: 10.1111/j.1755-148X.2011.00881.x (PMC3272348; doi:10.1111/j.1755-148X.2011.00881.x)
Supplement: Supplementary file 8 [file pcmr0024-0953-SD8.docx]

**Supplementary Figures**

**Figure S1.** Representative western blot of ETS-1 and its P-T38 phosphorylated fraction in normal human melanocytes (NHEM) and metastatic melanoma cell lines. P-T38/total ETS-1 ratios obtained by densitometric analysis are also shown.

**Figure S2**. A, Promoter luciferase assays obtained by cotransfecting different genomic fragments (as indicated in Figure 4A) in 293FT cells in presence or not of ETS-1. As controls, mutations of core nucleotides have been included. Data are representative of at least three independent experiments. *p<0.05. B, qRT-PCR analysis at different time points of ETS-1 mRNA (left) and miR-221/-222 (right). C, WB of ETS-1 and its P-T38-fraction, ERK1/2 and P-ERK1/2 in ETS-1 transfected 293FT cells. A375M is included as a positive control.

**Figure S3**. A, western blot analysis of ETS-1 and P-T38-ETS-1 in stably infected Me665/2/ETS-1 and A375M/TM4 melanoma cell lines compared with the corresponding empty vector transduced cells.

Nuclear and cytoplasmic extracts have been analyzed. Actin is shown as a loading control. B, left, schematic picture of ETS-1 protein and its dominant negative (TM) truncated form; right, WB analysis of TM-transduced-A375M (TM1 to TM6 represent six independent lentiviral infections). C, western blot analysis showing ETS-1 downregulation in Dsi-ETS-1 transfected A375M cells. Dsi-scrambled is included as a negative control and actin is shown as a loading control.

**Figure S4**. ETS-1 functional role in Me665/1 metastatic melanoma. qRT-PCR of ETS-1 and miR-222 in Dsi-ETS-1 transiently transfected Me665/1 cells (top). GAPDH and RNU6 were used to normalize. Invasion assay in the same Dsi-ETS-1 transfected cells (bottom). * p<0.05; ** p<0.01.

**Figure S5.** ETS-1/PLZF integrated function. qRT-PCR of ETS-1 and miR-222 in Dsi-ETS-1 transiently transfected A375M/PLZF cells (top). Relative invasion capabilities of A375M control, A375M/PLZF and A375M/PLZF/Dsi-ETS-1 cells (bottom). A Dsi-scrambled sequence was included as a negative control. * p<0.05; ** p<0.01.

**Figure S6.** Schematic representation of ETS-1-miR-222 regulatory circuitry in primary and metastatic melanomas. In primary melanomas lacking significant genomic alterations, as Me1007, high levels of inactive not phosphorylated ETS-1 repress miR-222 transcription. Conversely, in metastatic cells, as A375M, the constitutive activation of ERK1/2 induces ETS-1 phosphorylation at Thr38 and activates miR-222, possibly in cooperation with c-JUN. In turn, high amounts of miR-222 downregulates ETS-1, sustaining a high P-T38/total ETS-1 ratio.
